# Isolated Great Saphenous Vein Thrombosis in a Patient With COVID-19 Infection: Case Report and Review of the Literature

**DOI:** 10.7759/cureus.32196

**Published:** 2022-12-05

**Authors:** Abdalla Fadul, Eihab A Subahi, Nusiba Elamin, Elrazi A Ali, Wafa Mohammed, Sagda Sayed, Waail Rozi, Ahmed Akasha, Mohamed F. Elawad, ELMustafa Abdalla

**Affiliations:** 1 Internal Medicine, Hamad Medical Corporation, Doha, QAT; 2 Medical Education, Internal Medicine Fellowship Program, Hamad Medical Corporation, Doha, QAT; 3 Internal Medicine, One Brooklyn Health/Interfaith Medical Center, Brooklyn, USA; 4 Radiology, Hamad General Hospital, Doha, QAT

**Keywords:** venous thromboembolism, lower limb edema, isolated great saphenous vein thrombosis, thrombosis, covid 19

## Abstract

On 30 January 2020, the Director-General declared that the outbreak of coronavirus disease 2019 (COVID-19) caused by severe acute respiratory syndrome coronavirus 2 (SARS-CoV-2) constitutes a Public Health Emergency of International Concern (PHEIC), and on 11 March 2020, it was characterized as a pandemic. Since then, patients with COVID-19 infection are commonly reported to have an increased risk of thrombosis in various blood vessels due to hypercoagulability, blood stasis, and endothelial damage. In this study, we will present a case of a pregnant lady who was evaluated for right leg pain that started one week after having upper respiratory tract symptoms and COVID-19 infection confirmed by the COVID antigen (Ag) test. Further investigation with Doppler ultrasound (US) revealed complete right great saphenous vein thrombosis. This suggests that COVID-19 may lead to other adverse effects through damage to blood vessels.

## Introduction

Coronavirus disease 2019 (COVID-19) is an infectious disease caused by the severe acute respiratory syndrome coronavirus 2 (SARS-CoV-2) virus, a new strain of the coronavirus family. It was first reported in Wuhan, China, in December 2019. It has since spread to every country around the world. In January 2020, the World Health Organization (WHO) announced the outbreak of the disease as a "public health emergency of international concern" [1].

Primarily, COVID-19 infection results in respiratory complications. However, a COVID-19 infection may be associated with a hyper-coagulable state, which leads to microvascular and macrovascular arterial and venous thromboembolism (VTE) [2].

The true incidence of venous thromboembolic events (VTEs) in patients with COVID-19 remains unknown; however, it is becoming more apparent that VTEs such as pulmonary thromboembolism (PE) and deep vein thrombosis (DVTs) in patients with COVID-19 are associated with increased morbidity and mortality [3]. Despite the high number of cases reported in a patient with DVT and PE as a sequela of SARS-CoV-2 infection, to our knowledge, only a few cases have been reported in patients with superficial venous thrombosis. Our case report will describe one of the few cases presented with greater saphenous venous thrombosis after a diagnosis of COVID-19 infection.

## Case presentation

A 30-year-old, female, primigravida, 29 weeks of gestation, with no past medical history, came to the ED complaining of right leg pain and swelling for one week, associated with flu-like symptoms. She had no other complaint and denied any chest pain, shortness of breath (SOB), palpitation, or hemoptysis. Regarding her gynecology history, she was following up with Obstetrics and Gynecology (OBG) as a case of infertility for five years, had tried clomiphene seven months prior to conception, and had a history of hyperprolactinemia, which was treated and is now normal. Her surgical history was positive for appendectomy around 20 years ago. Upon further questioning, there is no personal or family history of VTE or miscarriages. She was a non-smoker or alcohol consumer.

Upon admission, her vitals were stable and afebrile, and, on examination, her BMI was 30.48. There was right leg swelling around the knee and thigh with warmness and redness. Examination of other systems was unremarkable. Basic labs, including complete blood count (CBC), renal function test (RFT), and liver function test (LFT), which were sent, were within normal limits, except the COVID antigen was positive. Chest X-ray (CXR) and electrocardiogram (ECG) were unremarkable.

**Figure 1:**
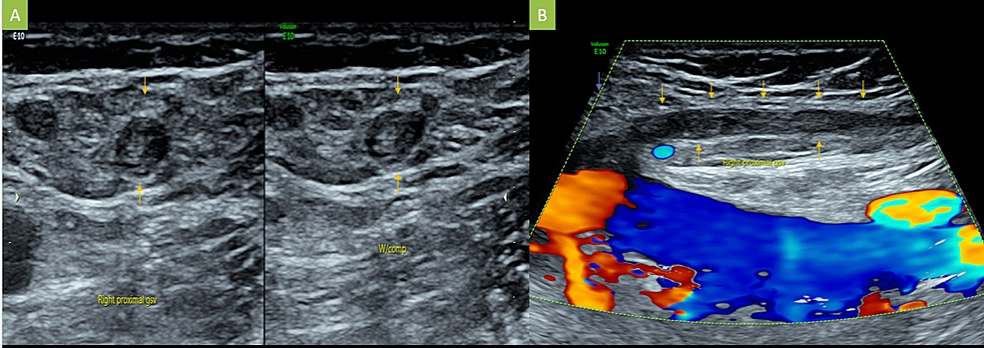
Gray-scale transverse US (A) and longitudinal venous Doppler examination (B) of the right medial thigh demonstrating right great saphenous vein with echogenic intraluminal thrombus, non-compressibility, and absent color Doppler flow, denoting thrombosis US: Ultrasound

US Doppler lower limb veins showed that the right great saphenous vein was thrombosed completely, there was an echogenic thrombus within its lumen, and it was not compressible on compression (Figure 1).

Our assessment Isolated great saphenous veins thrombosis (GSVT), uncomplicated, most likely due to COVID infection versus provoked by pregnancy. She was classified as moderate to high risk and started on a therapeutic enoxaparin dose of 80 mg BID SC during the pregnancy. Up to six weeks after delivery, she completed three months of therapeutic enoxaparin. She was discharged with internal medicine follow-up as an outpatient.

## Discussion

In our case, we presented a young pregnant lady diagnosed with COVID-19 infection, which was found to have right great saphenous vein thrombosis upon further investigation. She had common respiratory symptoms, including fever, throat pain, rhinorrhea, and cough. A COVID-19 polymerase chain reaction test (PCR) was done and came positive. She also presented with right leg swelling and pain, which was confirmed to be great saphenous venous thrombosis by Doppler ultrasound.

From asymptomatic to critically ill, the disease severity of COVID infections varies greatly. Since the epidemic, various problems have been reported. Severe COVID-19 can lead to crucial illness, with acute respiratory distress (ARDS) and multiorgan failure (MOF), eventually followed by intravascular coagulopathy [4]. In order to determine the prognosis of the condition, numerous common abnormal labs are checked and monitored while COVID-19 patients are in the hospital, including lymphocyte count dynamics and inflammatory indices (lactate dehydrogenase (LDH), CRP, and interleukin 6 (IL-6)), biomarkers such as high serum procalcitonin and ferritin, and blood hypercoagulability (elevated D-dimer levels, prothrombin time (PT) and aPTT prolongation, and fibrin-degradation products). Common comorbidities in hospitalized COVID-19 patients include prolonged bed rest due to illness, dehydration, an acute inflammatory state, the presence of other cardiovascular risk factors (such as diabetes, hypertension, and obesity), cardiovascular disease (such as coronary artery disease, history of ischemic stroke or peripheral artery disease), prior VTE history, and classical genetic thrombophilia potentially increase VTE risk. However, the COVID-19 disease has also been found to increase VTE risk, and it is observed that it strongly affects hemostasis and the hematopoietic system. It promotes a prothrombotic condition, and the significant number of serious thrombotic events that have been documented raises questions about its particular prothrombotic etiology [5].

The exact pathophysiology of thrombosis in COVID infection is not yet fully understood in the literature. The biomarkers of systemic inflammation, hypercoagulability brought on by disturbance of the renin-angiotensin-aldosterone system and a cytokine storm, and endothelial damage that alters the normal coagulation cascade are some of the proposed explanations for ischemic events [6]. In the vasculature of the lungs, spleen, brain, stomach, and periphery of COVID-19, microvascular and macrovascular thromboembolic or in situ thrombotic consequences have been noted [7]. A multicenter experience from northern Italy done by Marone EM et al. aimed to outline the main characteristics of DVT and pulmonary embolism (PE) in COVID-19 patients based on the experience of four high-volume COVID-19 hospitals in Northern Italy between 1 March and 25 April 2020. They concluded that DVT, thrombophlebitis, and PE are different aspects of COVID-19 procoagulant activity. They can arise regardless of the severity of respiratory impairment [8].

Given all of the aforementioned, patients with COVID infection are believed to be at a considerable risk of venous thromboembolism. We correlated this to our patient, who was in an outpatient setting, had no chronic illness or impaired coagulation profile, and was found to have great saphenous thrombosis pointing to COVID infection as the risk factor. Upon reviewing the literature, multiple cases of thrombosis related to COVID have been acknowledged, including DVT, PE, cerebral venous sinus thrombosis, superior mesenteric artery, and venous thrombosis [9]. However, few cases have been reported of great saphenous vein thrombosis. Young et al. conducted a systematic review and meta-analysis evaluating the incidence of pulmonary embolism and deep vein thrombosis in COVID-19 from 1 January 2020 to 15 June 2020. Twenty-seven studies with 3342 patients with COVID-19 were included, with the conclusion that PE and DVT occurred in 16.5% and 14.8% of patients with COVID-19, respectively [10].

Hesam-Shariati et al. reported a case of great saphenous vein thrombosis in a young man who was diagnosed with COVID pneumonia and was found to have evidence at the beginning of the greater saphenous vein of the right leg from distal to proximal on Doppler US. The patient was treated with anticoagulation without any complications afterward [11]. This is similar to our patient's diagnosis; however, our patient had COVID infection without pneumonia and had been treated in an outpatient setting, keeping in mind that no hospitalization is part of the thrombosis risk factor. In another case of Davoodi, Lotfollah et al. presented a case of lady presented with left leg pain, swelling, and redness as well as upper respiratory tract symptoms, who was diagnosed with COVID infection and was found to have thrombosis in the external iliac and left iliac veins up to the level of the bifurcation of the common iliac veins and at the superficial and small saphenous veins, treated with anticoagulation [12], which is different to our patient who presented only with great saphenous vein thrombosis without DVT.

## Conclusions

COVID-19 infection is considered a risk factor for thrombosis. The pathophysiology is not yet completely understood, but factors such as impaired coagulation, blood stasis, and endothelial damage have been linked. It is important to assess carefully for any signs of thrombosis in COVID-19 patients and to proceed with investigations and management accordingly. We are presenting this case to increase the awareness among physicians to include thrombosis in COVID-19 patients, even in unusual sites, in the differential diagnosis, aiming for early detection, better outcome, and preserving patients' lives.

As we all know, both pregnancy and COVID-19 infection are considered hypercoagulable states. From the literature, we can conclude that isolated great saphenous vein thrombosis alone is an unusual site of thrombosis during pregnancy but it is noted in some case reports to be linked to COVID-19 infection. In the end, we can put it the other way around: COVID-19 infection is the main cause or culprit and pregnancy is an additive risk factor.
